# Changes in the prevalence of U.S. adults using diet, exercise, pharmaceuticals and diet products for weight loss over time: Analysis of NHANES 1999–2018

**DOI:** 10.1371/journal.pone.0292810

**Published:** 2023-10-17

**Authors:** Jennifer L. Kuk, Simone B. Daniels, Chris I. Ardern, Rubin Pooni

**Affiliations:** School of Kinesiology and Health Sciences, York University, Toronto, ON, Canada; Gent University, BELGIUM

## Abstract

To examine changes in the use of diet, exercise, and pharmacological/diet product weight loss (WL) practices over time, and differences in these trends by sex and obesity status, data from the National Health and Examination Survey (NHANES Continuous 1999–2018) was used. The prevalence of diet, exercise and use of WL drugs and products over time were examined in men and women with and without obesity in a series of cross-sectional nationally representative samples (n = 43,020). Women and those with obesity were more likely to engage in WL practices over the past year, with an increased prevalence of WL efforts over time (38.4 to 43.2%). Amongst those who engaged in WL attempts, diet-related WL was most common (87–93%), followed by exercise-related WL (47–68%), whereas use of WL drugs and products was the least common (5–21%). There were modest differences in the prevalence of diet or exercise WL over time, with some differences by sex and obesity status. Most notable was the increase in the prevalence of exercise WL practices in women with obesity, with no differences among men or women without obesity. When examining specific types of diets, there were more clear differences in the adoption of diets over time, with the use of more traditional calorie/portion/fat restriction diets becoming less prevalent, and sugar/carbohydrate restriction becoming more prevalent over time (P<0.005). Changes over time in the use of diets were, were however, similar in men and women with and without obesity. Use of pharmacotherapy/diet products tended to decline in prevalence over time but was consistently highest in women with obesity. Thus, there are differences in the types of WL strategies individuals have employed over time, with variations in their popularity of use by sex and obesity status. However, the pattern of changes over time were quite similar in men and women with and without obesity.

## Introduction

As the obesity rate in the United States has increased over the past twenty years [1], so have the number of individuals who have engaged in weight loss [2]. Physical activity, diet, pharmacotherapy and weight loss products and supplements are commonly used weight loss approaches [3, 4], and examination of the pattern of changes in the popularity of these obesity management strategies has not been clearly examined. Further, differences in the engagement in these weight loss practices over time by sex and obesity status also warrants further investigation. Though it is clear that women are more likely to engage in weight loss than men [5–7] and in particular diet weight loss practices [5, 7, 8], whether the magnitude of this sex difference has changed over time is not clear. Similarly, individuals with obesity are more likely to attempt weight loss than individuals without obesity [2], but whether there are differences in the types of weight loss practices used among those attempting weight loss is not known. A recent study by Han et al. [9], reported the trends of diet and exercise weight loss in overweight versus normal weight adults, but did not stratify by sex. Further, the use of diet products and pharmaceutical weight loss products were not examined. Changes in the popularity of certain weight loss practices over time may occur for many reasons, including the influence of health professionals [10, 11] or popular media [12], which may have had differential effects on the popularity over time in men versus women with or without obesity. Thus, the objectives of this study were: 1) To examine the changes in the engagement of weight loss practices over time, and; 2) To determine whether the changes in the engagement of weight loss practices over time differed by sex and obesity status.

## Methods

### Survey methods

The publicly available National Health and Nutrition Examination Survey (NHANES) continuous surveys from years 1999 to 2018 were used for the current analyses (n = 101,316). NHANES is a series of nationally representative cross-sectional surveys of United States non-institutionalized civilians [13]. Participants gave informed written consent, and the study protocol was approved by the National Center for Health Statistics [14]. As this is an analysis of publicly available data, the current study did not require ethics approval from our institutional review board.

The analytical sample was restricted to participants aged 20 years and older (n = 55,081), with complete data for BMI, weight loss practices, smoking status and education. Participants were excluded if they were pregnant. Participants were also excluded if they had a BMI under <18.5 kg/m^2^ to limit the impact of individuals with eating disorders, leaving a final analytical sample of 43,020.

Questionnaires were used to assess participant demographics and weight loss practices [15]. Participants were asked “Was the change between your current weight and your weight a year ago intentional?” and “During the past 12 months, you tried to lose weight?”. If the response to either of those questions was ‘Yes’ (n = 15,218), individuals were considered to have attempted weight loss in the previous year and were asked about their engagement in specific weight loss practices. The following factors were consistently asked over all survey years: exercising, eating less food, eating low-calorie food, reducing fat intake, skipping meals, consuming diet foods, liquid diets, engagement in a weight loss program, use of diet products, drinking excess water or following a special weight loss diet. Engagement in diet-related weight loss practices was defined as the use of any of the following: eating less food, eating low-calorie food, reducing fat intake, skipping meals, consuming diet foods, liquid diets, special diets or drinking excess water, while the use of diet products and medications was defined as the use of prescription weight loss drugs, diet products or laxatives/vomiting. Data are presented in 4-year time intervals to improve the stability of estimates across the two NHANES surveys combined.

Sub-analyses were conducted on dietary variables collected only from 2005–2018: carbohydrate restriction, sugar restriction and consuming more fruits and vegetables. For these analyses, the first 6 survey years were collapsed into one category, and thereafter, similar 4-year increments in order to keep the survey year periods more comparable to the main analyses.

Standing height was measured to the nearest tenth of a centimeter (0.1 cm) using a stadiometer with a fixed vertical backboard and an adjustable head piece. Body weight was measured in kilograms using a digital weight scale. BMI was calculated using weight in kilograms divided by height in meters squared (kg/m^2^). No obesity (nOB) and obesity (OB) was defined using a BMI cut-off of 30 kg/m^2^.

### Statistical analysis

Continuous variables are presented as means with standard error, while categorical variables are presented as prevalence (%) with standard error. Descriptive characteristics are presented stratified by sex with changes across time assessed using linear regression.

Changes in the proportion of individuals who attempted weight loss over the last year was examined using regression with SAS survey procedures, with examination of sex-by-obesity-by-time 2- and 3-way interaction and main effects with adjustment for age, ethnicity, education status and smoking status. Because the changes over time were not consistently linear, time was examined as a continuous (presented in tables above figures) and categorical variable (Group comparison relative to 99–02 for the line graphs). Predicted least square adjusted mean proportions were computed to estimate differences in weight loss practices by sex, obesity status and survey year. Changes in the proportion of individuals engaging in certain weight management behaviours over time were assessed in only those who attempted weight loss over the last year, with adjustment for the same covariates as above using the same approach.

All analyses were weighted to be nationally representative of the United States population using SAS version 9.4 survey procedures (SAS Institute Inc., Cary, NC, USA) in accordance with the NHANES analytical guidelines [13, 16]. Statistical significance will be defined as p-value <0.05.

## Results

### Changes in weight loss attempt over time

Descriptive characteristics and obesity status for each four-year period are shown in **Table 1** for men and women. From 1999 to 2018, the proportion of individuals with obesity (29% to 39%) and attempting weight loss (35% to 43%) increased in both men and women (P < .0001) (**Table 1**).

**Table 1:** Participant characteristics over time.

**Male**	**99–02**		**03–06**		**07–10**		**11–14**		**15–18**		**p Trend**
**N**	**3796**		**3932**		**4886**		**4498**		**4337**		
**Age, year**	44.4	(0.4)	45.6	(0.6)	46.0	(0.4)	46.9	(0.5)	47.4	(0.4)	< .0001
**BMI, kg/m** ^**2**^	27.7	(0.1)	28.1	(0.2)	28.4	(0.1)	28.4	(0.1)	29.3	(0.2)	< .0001
**Obesity (%)**	25.7	(0.9)	29.7	(1.4)	32.0	(1.3)	32.3	(1.0)	38.6	(1.8)	< .0001
**Attempted WL (%)**	25.9	(0.8)	28.5	(1.1)	27.7	(1.0)	30.2	(1.0)	36.2	(1.3)	< .0001
**White Ethnicity (%)**	72.2	(1.6)	72.1	(2.1)	68.5	(2.5)	66.6	(2.6)	63.7	(2.3)	0.0009
**Smoker (%)**	27.8	(1.1)	28.2	(1.1)	24.4	(1.0)	22.9	(1.0)	20.4	(1.0)	< .0001
**Education (% > HS)**	52.2	(1.6)	54.5	(1.6)	54.7	(1.8)	60.4	(1.8)	60.0	(2.0)	0.0002
**Female**	**99–00**		**01–02**		**03–04**		**05–06**		**07–08**		**p Trend**
**N**	**3729**		**3712**		**4992**		**4595**		**4543**		
**Age, year**	46.5	(0.4)	48.2	(0.5)	48.2	(0.4)	48.7	(0.4)	49.6	(0.5)	< .0001
**BMI, kg/m** ^**2**^	28.3	(0.2)	28.3	(0.2)	28.6	(0.1)	29.1	(0.2)	29.5	(0.2)	< .0001
**Obesity (%)**	33.1	(1.1)	33.0	(1.2)	34.9	(0.7)	37.2	(1.3)	39.6	(1.1)	< .0001
**Attempted WL (%)**	44.0	(1.0)	48.8	(1.0)	44.9	(0.8)	47.4	(1.2)	49.8	(1.1)	0.0031
**White Ethnicity (%)**	70.4	(2.1)	71.7	(2.2)	69.3	(2.6)	65.5	(2.6)	62.8	(2.5)	0.0038
**Smoker (%)**	21.7	(1.1)	21.3	(0.9)	18.7	(0.8)	16.9	(1.0)	15.0	(0.7)	< .0001
**Education (% > HS)**	51.6	(1.6)	55.9	(1.3)	56.5	(1.2)	64.2	(1.8)	65.3	(1.5)	< .0001

Values presented are means or prevalences (standard error).

BMI = body mass index; WL = weight loss; HS = high school

### Changes in weight loss practices over time

**Fig 1:**
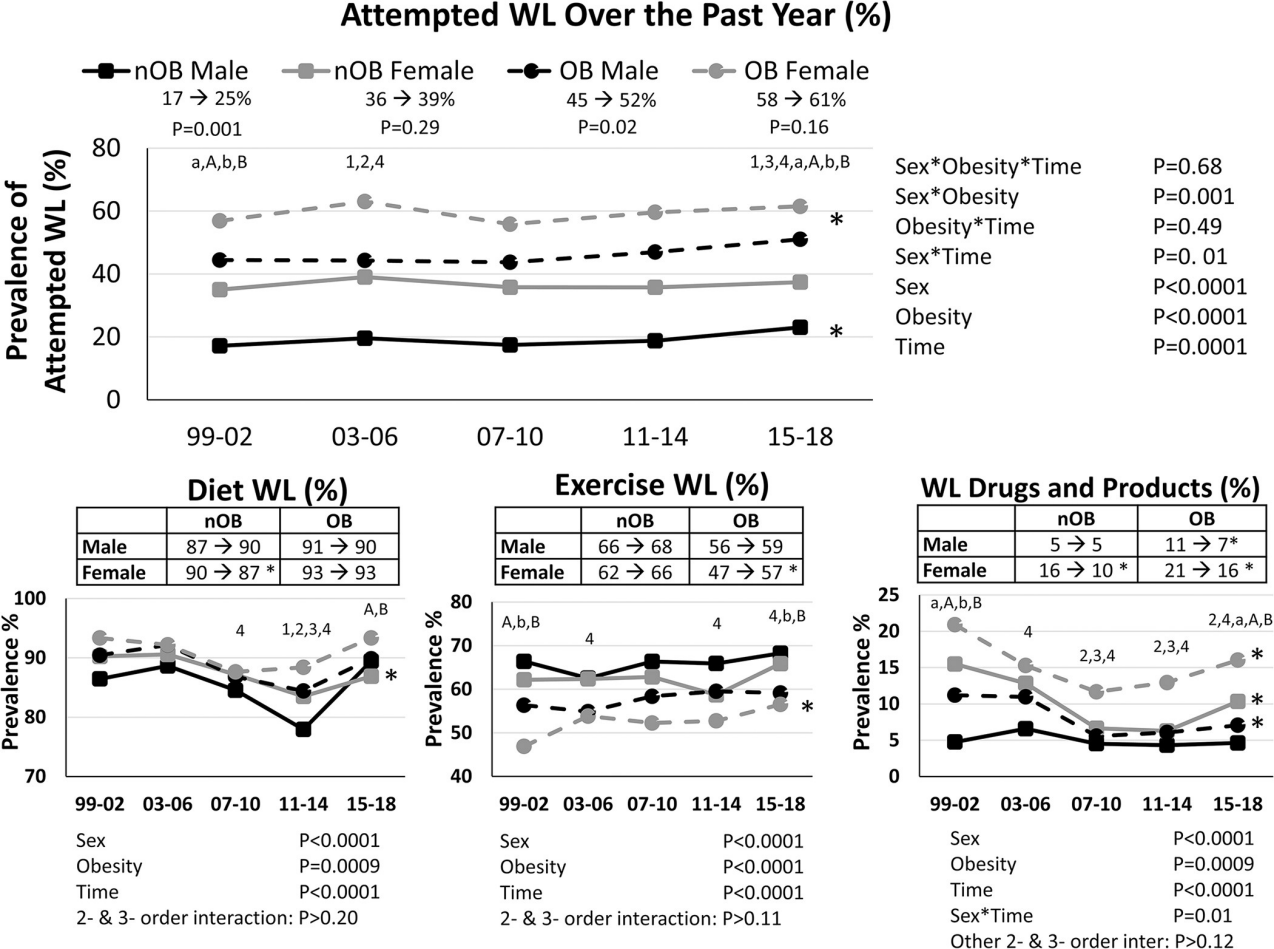
Prevalence of attempting weight loss (WL), and the types of WL approaches used from 1999–2018 in Men and Women with and without obesity. All models are adjusted for age, white ethnicity, education and smoking status. Top panel includes the full analytical sample (n = 43,020) and the bottom panels include only those who attempted weight loss over the last year (n = 15,218). nOB = no obesity; OB = obesity. * Significant trend over time within sex-obesity group (P<0.05). ^1^ P<0.05 compared to 1999–2002 for nOB male. ^2^ P<0.05 compared to 1999–2002 for nOB female. ^3^ P<0.05 compared to 1999–2002 for OB male. ^4^ P<0.05 compared to 1999–2002 for OB female. ^a^ P<0.05 sex difference within nOB. ^A^ P<0.05 sex difference within OB. ^b^ P<0.05 for OB diff within male. ^B^ P<0.05 for OB diff within female.

When stratified by sex and obesity status, and adjusted for age, ethnicity, education status and smoking status, the prevalence of men who attempted weight loss increased over time, regardless of weight status (nOB: 17 to 25%; OB: 45 to 52%, P< 0.02), but with no changes over time in prevalence of weight loss attempts in females (nOB: 36 to 39%; OB: 58 to 61%, P> 0.16; **Fig 1**). As expected, those with obesity had a greater prevalence of attempted weight loss than individuals without obesity (P<0.0001). Within a given obesity class, women had a greater prevalence of weight loss attempts than men (P<0.0001). On average, individuals who attempted weight loss reported engaging in approximately three weight loss practices (nOB Men: 2.63(0.04); nOB Women: 2.84(0.03); OB Men: 2.75(0.04); OB Women: 3.1(0.04)) within the past year, with no significant differences over time.

**Fig 2:**
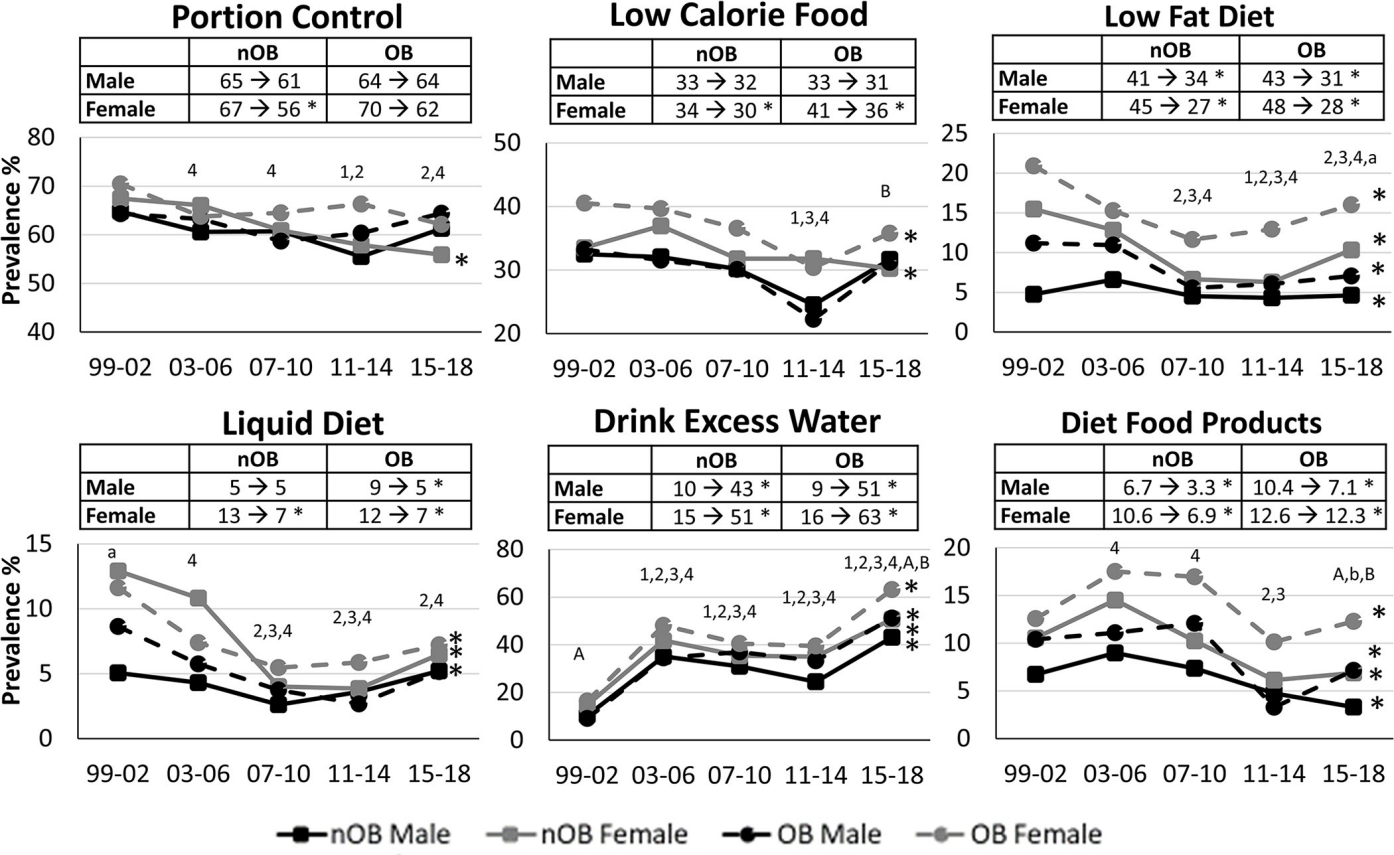
Prevalence of various types of diet weight loss (WL) approaches used in those attempting WL from 1999–2018 in Men and Women with and without obesity. All models are adjusted for age, white ethnicity, education and smoking status (n = 15,218). nOB = no obesity; OB = obesity; WL = weight loss. * Significant trend over time within sex-obesity group (P<0.05). ^1^ P<0.05 compared to 1999–2002 for nOB male. ^2^ P<0.05 compared to 1999–2002 for nOB female. ^3^ P<0.05 compared to 1999–2002 for OB male. ^4^ P<0.05 compared to 1999–2002 for OB female. ^a^ P<0.05 sex difference within nOB. ^A^ P<0.05 sex difference within OB. ^b^ P<0.05 for OB diff within male. ^B^ P<0.05 for OB diff within female.

**Fig 3:**
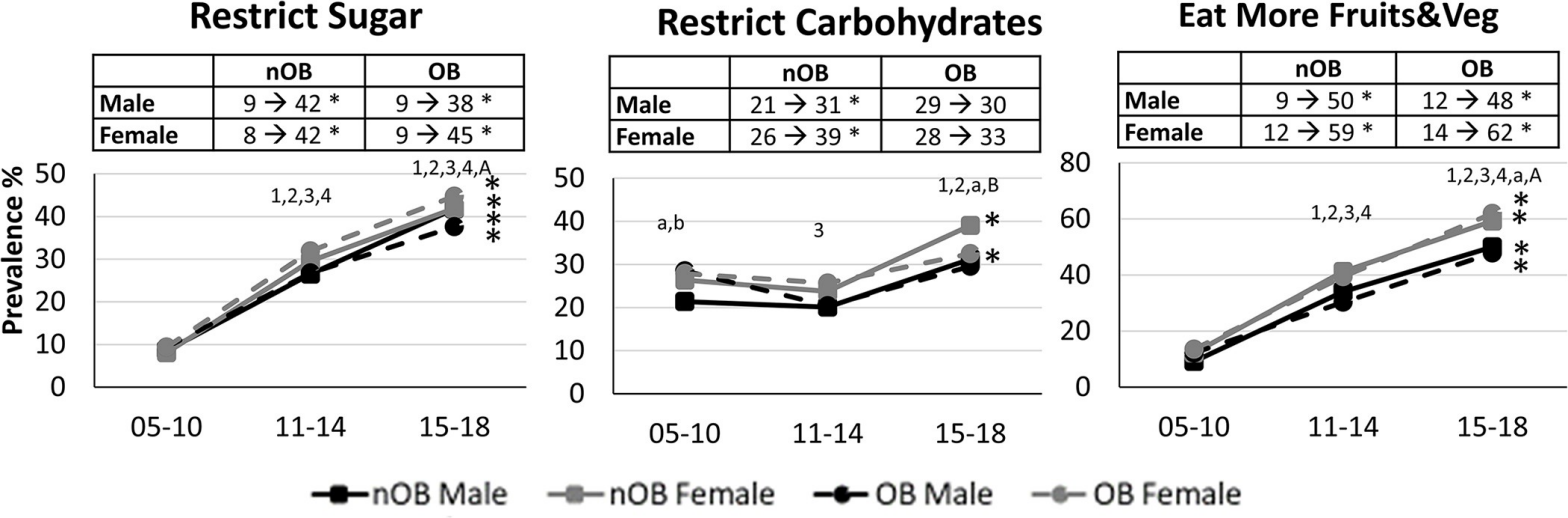
Prevalence of various types of diet weight loss (WL) approaches used in those attempting WL from 1999–2018 in Men and Women with and without obesity. All models are adjusted for age, white ethnicity, education and smoking status (n = 15,218). nOB = no obesity; OB = obesity; WL = weight loss. * Significant trend over time within sex-obesity group (P<0.05). ^1^ P<0.05 compared to 2002–2010 for nOB male. ^2^ P<0.05 compared to 2002–2010 for nOB female. ^3^ P<0.05 compared to 2002–2010 for OB male. ^4^ P<0.05 compared to 2005–2010 for OB female. ^a^ P<0.05 sex difference within nOB. ^A^ P<0.05 sex difference within OB. ^b^ P<0.05 for OB diff within male. ^B^ P<0.05 for OB diff within female.

When broken down by weight loss practices, there were clear differences in the prevalence and trends in use over time by sex and obesity status (**Fig 1**). In general, diet-related weight loss practices were the most common form of attempted weight loss, while use of weight loss medications and products were the least common. Women and individuals with obesity were more likely to have reported engaging in diet weight loss as compared to men and nOB individuals (P<0.001). Most sex and obesity groups demonstrated no significant change in the prevalence of individuals engaged in diet weight loss over time. The only exception to this was a modest 3% decline over time in in the prevalence of diet weight loss engagement within nOB women (P = 0.009, **Fig 1**). However, when examining specific types of diet weight loss methods, there were clear sex and obesity differences in the pattern of use of the different types of diets over time (**Figs 2 and 3**). While the prevalence of some of the more traditional diets, such as portion control, low calorie diets, low fat diets, liquid diets, and diet food products, tended to decline over time, the prevalence of other diet weight loss strategies, such as drinking more water, restricting sugar intake and eating more fruits and vegetables, dramatically increased in all sex-obesity groups (P<0.0001), while use of carbohydrate restrictive diets only increased in nOB men and women (P<0.005).

Exercise weight loss was less prevalent than diet weight loss, and more common than the use of weight loss medications and products. Men and nOB individuals were more likely to engage in exercise weight loss (P<0.0001). Over time, there was an increased prevalence in OB women engaging in exercise weight loss (P = 0.02), but no significant changes in men or nOB women (P>0.3, **Fig 1)**.

Use of weight loss drugs and products decreased over time in all sex-OB groups (P<0.05, **Fig 1**), except for nOB men wherein the prevalence remained relatively consistent (~5%) across the study period (P = 0.99). When prescription weight loss and diet products/supplements were examined separately, there was a general trend for decreased usage in those with obesity, but with the low prevalence of weight loss drug use, these differences did not reach statistical significance (nOB Female: 2.2 to 3.3%; OB Female: 6.9 to 5.9%; nOB Male: 0.1 to 0.3%; OB Male: 2.8 to 1.6%, P>0.22). Only a small proportion (4 to 14%) of individuals reported use of diet products to lose weight across all time points and subgroups. A modest drop in the use of diet products and supplements in nOB women (13% to 7%, P = 0.0008) and men with obesity (7.4% to 5.0%, P = 0.03), was observed, with no significant differences in the other subgroups (P>0.05).

## Discussion

The aim of this study was to evaluate trends in engagement of weight management practices over time. Over time, there was an increased proportion of men who attempted to lose weight, but no difference in women, regardless of obesity status. Amongst individuals trying to lose weight, there were either no, or only modest changes in the proportion of individuals using dietary weight loss methods. Over time, however, there was an increase in the prevalence of exercise weight loss engagement and a surprising reduction in the use of weight loss drugs and weight loss products over time, given their already low prevalence of use. Although there were sex and obesity differences in the absolute prevalence of engagement in the various weight management strategies, the pattern of changes over time were generally similar in men and women with or without obesity, with the notable exception being a larger increase in exercise weight loss in women with obesity over time than other sex-OB groups.

### Weight loss intentions

The prevalence of individuals engaging in weight loss approaches were stable in women, and increased by 6 to 7% in men, regardless of obesity status. Nevertheless, women with obesity remained the most likely to have recently attempted weight loss. This is consistent with other literature [2] and is reflected in the high prevalence of women with obesity in many weight management programs [17, 18]. During 2015–2018, over 60% of women with obesity attempted to lose weight over the last year, whereas ~40% of women without obesity also attempted to lose weight. On the other hand, men without obesity were the least likely to have attempted weight loss over the past year. Nevertheless, 1 in 4 men without obesity engaged in weight loss at the end of our survey period in 2018. This is not a trivial amount and reflects the high and increasing prevalence of weight loss behaviours in the U.S, even among those who have not yet developed obesity. Amongst those with obesity, 52% of men and 61% of women attempted weight loss in 2015–2018. Older clinical weight management guidelines recommend that all individuals with a BMI over 30 kg/m^2^ be prescribed weight loss [19], while more novel approaches such as the Edmonton Obesity Staging System, suggest that weight loss is warranted for only individuals with a BMI greater than 30 kg/m^2^ if they have obesity-related comorbidities, which translates into approximately 75% of individuals with a BMI over 30 kg/m^2^ [20]. Thus, the proportion of individuals with a BMI over 30 kg/m^2^ attempting weight loss, appears to be less than what would be expected given either approach.

### Exercise weight loss

Exercise is well known to be associated with health benefits, and data from the Behavioral Risk Factor Surveillance System (BRFSS) suggests that more adults engaged in exercise from 2001 to 2007 [21]. Over time, there have been several significant physical activity promotion campaigns that may have led to the increased engagement in exercise. However, when specifically examining changes in the prevalence of exercise for the purposes of weight loss, we only observed an increase in exercise weight loss in women with obesity. This may in part reflect the movement towards increasing acceptance towards women’s bodies [22], as body image and exercise are positively correlated [23]. Indeed, those who perceive themselves as overweight were more likely to exercise for weight loss [9]. Despite the increase over time, women with obesity remained the least likely at all time points to engage in exercise for the purposes of weight loss. However, this may be expected considering that female sex [24] and obesity [25] are both associated with less exercise associated weight loss. In fact, exercise in the absence of caloric restriction is typically associated with weight losses of only 2 kg [26]. Given this, it might be surprising that over half of individuals attempted to lose weight by means of exercise in the past year. As the health benefits of exercise extend beyond simply losing weight [26] or better weight loss maintenance [27], further increases in exercise adoption through a reduction in barriers that prevent individuals from engaging in exercise may have wide-reaching health benefits.

### Diet weight loss

The overall prevalence of individuals engaging in diet weight loss fluctuated over time, but has remained above 80% for most time points and was the most common form of weight loss intervention used. When the specific types of diet were examined, it was clear that the popularity of certain diet interventions has fluctuated over time, with many diets declining in popularity over time, while others have increased in prevalence. Dietary approaches, such as portion control, low calorie, liquid and low-fat diets have been used for weight loss and researched for several decades [19, 28–31]. However, data from this analysis indicates that the popularity of these dietary weight loss approaches may be declining in popularity. Though several categories of weight loss diets were examined in the current study, the list is not extensive, with examination of specific dietary approaches, such as high protein (i.e., Atkins or Ketogenic diets) or the Mediterranean diet, being notable omissions. Characteristics of these diets include restriction of sugar and carbohydrate intake and/or increasing fruit and vegetable consumption which were only examined beginning in 2005. To this end, two notable patterns were observed. First, by the end of the survey period, 30–45% of adults had attempted restricting sugar and/or carbohydrates, reflecting the recent popularity of ketogenic dietary weight loss approaches. Second, the Mediterranean diet (characterized by high consumption of fresh produce [32]) and the use of increasing fruit and vegetable consumption for weight loss increased five-fold between 2005–2010 and 2015–2018.

Though individuals were not asked directly about their specific engagement in any diet weight loss intervention, 80–95% of individuals engaging in weight loss reported using one of the diets examined in this study. Thus, it is unlikely that inclusion of additional diets would have led to a substantially greater proportion of dietary weight loss engagement. Similar to the current analysis, others have noted that women were more likely to engage in caloric restriction than men [5]. Furthermore, the engagement of diet weight loss practices was most common in women with obesity, with obesity differences being more clearly demonstrated in females than males. It is interesting to note that the differences in the types of dietary approaches used and their changes over time were quite similar between men and women and by obesity status, suggesting that popular trends in weight loss practice may be more ubiquitous in their adoption. Though it is clear that there are variations in the popularity of certain diets over time [2, 33], there is no one-size-fits-all dieting approach. While there are some potential differences in short term weight loss, the long-term differences in weight loss between most dietary approaches are quite modest [34]. Thus, the use of dietary weight loss strategies is highly prevalent among those attempting to lose weight, particularly women with obesity, but the specific dietary approaches used have varied over time.

### Prescription weight loss pharmaceuticals, diet pills and supplements or weight loss products

The use of non-lifestyle-based weight loss methods, such as prescription weight loss medications, diet pills, supplements and other diet products, are far less prevalent than lifestyle approaches and decreased over time. The use of prescription weight loss medications was most common in women with obesity, but with a prevalence of less than 10% for women with obesity and less than 2% for men with obesity, this is far less than what would be observed for other chronic diseases. Indeed, over half to three quarters of individuals with hypertension report taking prescription medications [35], and nearly 90% of adults with diabetes report using pharmaceutical intervention [36]. To compound issues, the 1- and 2-year compliance for taking obesity medications are also far lower than medications used for other chronic conditions [37]. The lower long-term compliance is likely a reflection of high cost due to the lack of insurance coverage, low prescription rates and/or and side effects [37–39] that are often associated with obesity medications. Given that obesity is now recognized as a chronic disease [40], and effective pharmacological options such as Wegovy [41] are now available, focus on increasing the appropriate use of weight loss pharmaceuticals in those with obesity is needed.

The use of diet products fluctuated between 4 to 14% depending on the sex, obesity status and time point. Unfortunately, it is unclear what specific diet products or supplements were used by the participants. Although the use of diet supplements and products was much lower than lifestyle approaches (5 to 15% versus 50 to 90%), it was also much greater than prescription weight loss medications (<6%). The ever-evolving, new array of diet products that are developed and bought are advertised as relatively inexpensive, safe and effective obesity treatments, which may explain their sustained usage over time. This is problematic, however, as there is a shortage of evidence on the efficacy of many of the diet supplements and weight loss products on the market [42, 43]. Further, some herbal and dietary supplements have been linked with adverse health effects [42, 44]. Their sustained use over time likely reflects the general lack of evidence-based lifestyle or pharmacological options that induce the magnitude of weight loss desired by most individuals with obesity or their relatively higher cost [45].

### Strengths and limitations

There are several strengths and limitations worth mentioning. The use of a large, nationally representative sample of the U.S. population, allowed for the tracking of weight loss practices over time. Notwithstanding the above, our analysis involves the examination of sequential cross-sectional survey cycles, and the length of time the individuals engaged in weight loss practices or the success of these interventions was not assessed. However, as most individuals regain weight shortly after lifestyle weight loss, it is likely that the long term weight reduction for most would be modest at best [46]. Further, details such as body image or the severity of the intervention were also not captured (i.e., degree of fat or calorie restriction, the intensity and time spent engaging in exercise, etc.) and whether they were used concurrently or sequentially is not known. Finally, the issue of reporting bias cannot be precluded.

## Conclusion

In summary, this study observed changing patterns of engagement in weight loss practices in a representative sample of U.S. adults over time. Further research is needed to explore the reasons for these changing trends, and how the public can be informed and adopt the latest evidence-based recommendations for obesity prevention and management.

## Supporting information

S1 Checklist *PLOS ONE* clinical studies checklist. (DOCX)

S2 Checklist STROBE statement—checklist of items that should be included in reports of *cohort studies*. (DOCX)
