# Supplementary material for: Changes in the prevalence of U.S. adults using diet, exercise, pharmaceuticals and diet products for weight loss over time: Analysis of NHANES 1999–2018
Source: PLoS One. 2023 Oct 17;18(10):e0292810. doi: 10.1371/journal.pone.0292810 (PMC10581481; doi:10.1371/journal.pone.0292810)
Supplement: Attachment — Submitted filename: NHANES .docx [file pone.0292810.s003.docx]

**Review**

I reviewed the manuscript entitled “Changes in the Prevalence of U.S. Adults Using Diet, Exercise, Pharmaceuticals, and Diet Products for Weight Loss Over Time: Analysis of NHANES 1999-2018.”. The manuscript is in the scope of the journal, but certain shortcomings should be addressed before the article could be published:

•	There are several language and grammar mistakes in the manuscript. A thorough editing is required. WL is the abbreviation mentioned for weight loss, though in some parts it is also used instead of WL exercises making the phrases confusing

•	In the abstract part: a sample size should be added.

•	Various factors motivate individuals to get engaged in various WL plans, being obese alone is not necessarily the main factor. Body image also plays a critical role which has been neglected in this study. Moreover, underlying diseases are the motivation for such changes, these have also been ignored. In brief, the factors mentioned in this study are important but not sufficient alone. The authors should explain how they have managed to overcome these confounding factors, or else this study is biased.
