# Supplementary material for: Changes in the prevalence of U.S. adults using diet, exercise, pharmaceuticals and diet products for weight loss over time: Analysis of NHANES 1999–2018
Source: PLoS One. 2023 Oct 17;18(10):e0292810. doi: 10.1371/journal.pone.0292810 (PMC10581481; doi:10.1371/journal.pone.0292810)
Supplement: Attachment — Submitted filename: Response to Reviewers.docx [file pone.0292810.s004.docx]

**Reviewer and Editor Comments**

We thank the Reviewers and Editor for your comments. Please see our point by point responses below each of the Editor and Reviewer comments.

**Editor Comments**

1. Please ensure that your manuscript meets PLOS ONE's style requirements, including those for file naming. The PLOS ONE style templates can be found at

<https://journals.plos.org/plosone/s/file?id=wjVg/PLOSOne_formatting_sample_main_body.pdf> and

<https://journals.plos.org/plosone/s/file?id=ba62/PLOSOne_formatting_sample_title_authors_affiliations.pdf>

**Response** - We have revised the abstract and removed headings to meet the formatting requirements. Please let us know if we have missed something else.

2. Thank you for stating the following financial disclosure:

“The authors received no specific funding for this work.  Institutional funding from York University was used to support this manuscript.  York University did not have a role in the manuscript writing editing approval or decision to publish.”

At this time, please address the following queries:

a) Please clarify the sources of funding (financial or material support) for your study. List the grants or organizations that supported your study, including funding received from your institution.

b) State what role the funders took in the study. If the funders had no role in your study, please state: “The funders had no role in study design, data collection and analysis, decision to publish, or preparation of the manuscript.”

c) If any authors received a salary from any of your funders, please state which authors and which funders.

d) If you did not receive any funding for this study, please state: “The authors received no specific funding for this work.”

Please include your amended statements within your cover letter; we will change the online submission form on your behalf.

**Response** - We have revised the funding statement to: “The authors received no specific funding for this work.”

3. We noted in your submission details that a portion of your manuscript may have been presented or published elsewhere. [This manuscript or its essence has not been published as a peer-review but a prior version of this manuscript was published as part of Simone Daniels’ MSc thesis at York University. This manuscript includes additional data from the latest NHANES survey (2017-2018), and with differing analyses and results (<https://yorkspace.library.yorku.ca/xmlui/bitstream/handle/10315/38812/Daniels_Simone_B_2021_Masters.pdf?sequence=2&isAllowed=y>)] Please clarify whether this [conference proceeding or publication] was peer-reviewed and formally published. If this work was previously peer-reviewed and published, in the cover letter please provide the reason that this work does not constitute dual publication and should be included in the current manuscript.

**Response** - The thesis was not peer reviewed, and is published online by the university library. This is standard practice at most if not all Canadian Universities. Although the general topic is the same, the manuscript is substantially different from the thesis paper in its analyses and conclusions. Thus, we feel that this would not constitute dual publication.

4. We note that you have included the phrase “data not shown” in your manuscript. Unfortunately, this does not meet our data sharing requirements. PLOS does not permit references to inaccessible data. We require that authors provide all relevant data within the paper, Supporting Information files, or in an acceptable, public repository. Please add a citation to support this phrase or upload the data that corresponds with these findings to a stable repository (such as Figshare or Dryad) and provide and URLs, DOIs, or accession numbers that may be used to access these data. Or, if the data are not a core part of the research being presented in your study, we ask that you remove the phrase that refers to these data.

**Response** – We have removed that phrase in the manuscript and have added the results to the manuscript.

**Review Comments to the Author**

Reviewer #1: There are several language and grammar mistakes in the manuscript. A thorough editing is required. WL is the abbreviation mentioned for weight loss, though in some parts it is also used instead of WL exercises making the phrases confusing
**Response** – We apologize, but the abbreviation WL is only used in the abstract and tables/figures, and is meant to only mean weight loss. I have asked a colleague to edit, and have made several changes to hopefully improve the clarity of our writing.

• In the abstract part: a sample size should be added.

**Response** – added.

• Various factors motivate individuals to get engaged in various WL plans, being obese alone is not necessarily the main factor. Body image also plays a critical role which has been neglected in this study. Moreover, underlying diseases are the motivation for such changes, these have also been ignored. In brief, the factors mentioned in this study are important but not sufficient alone. The authors should explain how they have managed to overcome these confounding factors, or else this study is biased.

**Response** – We complete agree with the reviewer that body image and weight loss intention likely play a large role in weight management behaviours. However, if you take the perspective of who should be prescribed weight loss, then guidelines normally suggest one of two approaches: 1) anyone over BMI of 30 kg/m^2^ or 2) BMI above 25 with comorbidities. Using approaches such as the Edmonton obesity staging system (EOSS), over 95% of individuals with a BMI greater than 30 kg/m2 have some sort of obesity related complication. Thus, our use of BMI alone would encompass the majority of individuals who would be prescribed weight loss in the clinical setting. We have added this omission to the discussion (page 15) and the limitations.

**Reviewer #2:**

As a descriptive article, it had acceptable analysis information, but it may be better to express the practical goals of this study more clearly.
**Response** – Thank you. We have revised the manuscript to more clearly express the study objectives at the end of the introduction.

According to the fact that all the participants had informed consent for the use of their information from the beginning, it is reasonable to assume that there was no need for review by the ethics committee and the study is acceptable from the ethical point of view.

#### Response – Be assured that we followed the ethical guidelines in Canada. According to the Tri-Council Policy Statement: Ethical Conduct for Research Involving Humans (2022), Article 2.2:

“Research does not require REB review when it relies exclusively on information that is:

a. publicly available through a mechanism set out by legislation or regulation and that is protected by law; or
b. in the public domain and the individuals to whom the information refers have no reasonable expectation of privacy.“

Our research falls under point b: data in the public domain, and thus did not require REB review.
