# Supplementary material for: Changes in the prevalence of U.S. adults using diet, exercise, pharmaceuticals and diet products for weight loss over time: Analysis of NHANES 1999–2018
Source: PLoS One. 2023 Oct 17;18(10):e0292810. doi: 10.1371/journal.pone.0292810 (PMC10581481; doi:10.1371/journal.pone.0292810)
Supplement: Attachment — Submitted filename: Review final.docx [file pone.0292810.s005.docx]

**Review**

I reviewed the manuscript entitled “**Changes in the Prevalence of U.S. Adults using Diet, Exercise, Pharmaceuticals and Diet Products for Weight Loss Over Time: Analysis of NHANES 1999-2018**”. The manuscript is in the scope of the journal and can be published after a precise evaluation of the responses and changes.
