# Supplementary material for: Changes in the prevalence of U.S. adults using diet, exercise, pharmaceuticals and diet products for weight loss over time: Analysis of NHANES 1999–2018
Source: PLoS One. 2023 Oct 17;18(10):e0292810. doi: 10.1371/journal.pone.0292810 (PMC10581481; doi:10.1371/journal.pone.0292810)
Supplement: Peer review history

# Peer review: Decision Letter 0

**DOI:** 10.1371/journal.pone.0292810.r001

**Authors:** Patricia Khashayar

31 Jul 2023

PONE-D-23-14042Changes in the Prevalence of U.S. Adults using Diet, Exercise, Pharmaceuticals and Diet Products for Weight Loss Over Time: Analysis of NHANES 1999-2018.PLOS ONE

Dear Dr. Kuk,

Thank you for submitting your manuscript to PLOS ONE. After careful consideration, we feel that it has merit but does not fully meet PLOS ONE’s publication criteria as it currently stands. Therefore, we invite you to submit a revised version of the manuscript that addresses the points raised during the review process.

==============================

Apart from language editing and certain comments raised by the reviewers, the  comment raised regarding the factors affecting WL plans and the fact that only some of them was addressed in this article is quite important. Please address this comment specifically before resubmitting the article

==============================

Please submit your revised manuscript by Sep 14 2023 11:59PM. If you will need more time than this to complete your revisions, please reply to this message or contact the journal office at plosone@plos.org. When you're ready to submit your revision, log on to https://www.editorialmanager.com/pone/ and select the 'Submissions Needing Revision' folder to locate your manuscript file.

Please include the following items when submitting your revised manuscript:

- A rebuttal letter that responds to each point raised by the academic editor and reviewer(s). You should upload this letter as a separate file labeled 'Response to Reviewers'.
- A marked-up copy of your manuscript that highlights changes made to the original version. You should upload this as a separate file labeled 'Revised Manuscript with Track Changes'.
- An unmarked version of your revised paper without tracked changes. You should upload this as a separate file labeled 'Manuscript'.

If you would like to make changes to your financial disclosure, please include your updated statement in your cover letter. Guidelines for resubmitting your figure files are available below the reviewer comments at the end of this letter.

If applicable, we recommend that you deposit your laboratory protocols in protocols.io to enhance the reproducibility of your results. Protocols.io assigns your protocol its own identifier (DOI) so that it can be cited independently in the future. For instructions see: https://journals.plos.org/plosone/s/submission-guidelines#loc-laboratory-protocols. Additionally, PLOS ONE offers an option for publishing peer-reviewed Lab Protocol articles, which describe protocols hosted on protocols.io. Read more information on sharing protocols at https://plos.org/protocols?utm_medium=editorial-email&utm_source=authorletters&utm_campaign=protocols.

We look forward to receiving your revised manuscript.

Kind regards,

Patricia Khashayar

Academic Editor

PLOS ONE

Journal requirements:

When submitting your revision, we need you to address these additional requirements.

1. Please ensure that your manuscript meets PLOS ONE's style requirements, including those for file naming. The PLOS ONE style templates can be found at

https://journals.plos.org/plosone/s/file?id=wjVg/PLOSOne_formatting_sample_main_body.pdf and

https://journals.plos.org/plosone/s/file?id=ba62/PLOSOne_formatting_sample_title_authors_affiliations.pdf

2. Thank you for stating the following financial disclosure:

“The authors received no specific funding for this work.  Institutional funding from York University was used to support this manuscript.  York University did not have a role in the manuscript writing editing approval or decision to publish.”

At this time, please address the following queries:

a) Please clarify the sources of funding (financial or material support) for your study. List the grants or organizations that supported your study, including funding received from your institution.

b) State what role the funders took in the study. If the funders had no role in your study, please state: “The funders had no role in study design, data collection and analysis, decision to publish, or preparation of the manuscript.”

c) If any authors received a salary from any of your funders, please state which authors and which funders.

d) If you did not receive any funding for this study, please state: “The authors received no specific funding for this work.”

Please include your amended statements within your cover letter; we will change the online submission form on your behalf.

3. We noted in your submission details that a portion of your manuscript may have been presented or published elsewhere. [This manuscript or its essence has not been published as a peer-review but a prior version of this manuscript was published as part of Simone Daniels’ MSc thesis at York University. This manuscript includes additional data from the latest NHANES survey (2017-2018), and with differing analyses and results (https://yorkspace.library.yorku.ca/xmlui/bitstream/handle/10315/38812/Daniels_Simone_B_2021_Masters.pdf?sequence=2&isAllowed=y)] Please clarify whether this [conference proceeding or publication] was peer-reviewed and formally published. If this work was previously peer-reviewed and published, in the cover letter please provide the reason that this work does not constitute dual publication and should be included in the current manuscript.

4. We note that you have included the phrase “data not shown” in your manuscript. Unfortunately, this does not meet our data sharing requirements. PLOS does not permit references to inaccessible data. We require that authors provide all relevant data within the paper, Supporting Information files, or in an acceptable, public repository. Please add a citation to support this phrase or upload the data that corresponds with these findings to a stable repository (such as Figshare or Dryad) and provide and URLs, DOIs, or accession numbers that may be used to access these data. Or, if the data are not a core part of the research being presented in your study, we ask that you remove the phrase that refers to these data.

[Note: HTML markup is below. Please do not edit.]

Reviewers' comments:

Reviewer's Responses to Questions

**Comments to the Author**

1. Is the manuscript technically sound, and do the data support the conclusions?

The manuscript must describe a technically sound piece of scientific research with data that supports the conclusions. Experiments must have been conducted rigorously, with appropriate controls, replication, and sample sizes. The conclusions must be drawn appropriately based on the data presented. 

Reviewer #1: Yes

Reviewer #2: Yes

**********

2. Has the statistical analysis been performed appropriately and rigorously? 

Reviewer #1: Yes

Reviewer #2: Yes

**********

3. Have the authors made all data underlying the findings in their manuscript fully available?

The PLOS Data policy requires authors to make all data underlying the findings described in their manuscript fully available without restriction, with rare exception (please refer to the Data Availability Statement in the manuscript PDF file). The data should be provided as part of the manuscript or its supporting information, or deposited to a public repository. For example, in addition to summary statistics, the data points behind means, medians and variance measures should be available. If there are restrictions on publicly sharing data—e.g. participant privacy or use of data from a third party—those must be specified.

Reviewer #1: No

Reviewer #2: Yes

**********

4. Is the manuscript presented in an intelligible fashion and written in standard English?

PLOS ONE does not copyedit accepted manuscripts, so the language in submitted articles must be clear, correct, and unambiguous. Any typographical or grammatical errors should be corrected at revision, so please note any specific errors here.

Reviewer #1: No

Reviewer #2: Yes

**********

5. Review Comments to the Author

Please use the space provided to explain your answers to the questions above. You may also include additional comments for the author, including concerns about dual publication, research ethics, or publication ethics. (Please upload your review as an attachment if it exceeds 20,000 characters)

Reviewer #1: There are several language and grammar mistakes in the manuscript. A thorough editing is required. WL is the abbreviation mentioned for weight loss, though in some parts it is also used instead of WL exercises making the phrases confusing

• In the abstract part: a sample size should be added.

• Various factors motivate individuals to get engaged in various WL plans, being obese alone is not necessarily the main factor. Body image also plays a critical role which has been neglected in this study. Moreover, underlying diseases are the motivation for such changes, these have also been ignored. In brief, the factors mentioned in this study are important but not sufficient alone. The authors should explain how they have managed to overcome these confounding factors, or else this study is biased.

Reviewer #2: As a descriptive article, it had acceptable analysis information, but it may be better to express the practical goals of this study more clearly.

According to the fact that all the participants had informed consent for the use of their information from the beginning, it is reasonable to assume that there was no need for review by the ethics committee and the study is acceptable from the ethical point of view.

**********

6. PLOS authors have the option to publish the peer review history of their article (what does this mean?). If published, this will include your full peer review and any attached files.

If you choose “no”, your identity will remain anonymous but your review may still be made public.

**Do you want your identity to be public for this peer review?** For information about this choice, including consent withdrawal, please see our Privacy Policy.

Reviewer #1: No

Reviewer #2: **Yes: **zahrapanahi

**********

[NOTE: If reviewer comments were submitted as an attachment file, they will be attached to this email and accessible via the submission site. Please log into your account, locate the manuscript record, and check for the action link "View Attachments". If this link does not appear, there are no attachment files.]

While revising your submission, please upload your figure files to the Preflight Analysis and Conversion Engine (PACE) digital diagnostic tool, https://pacev2.apexcovantage.com/. PACE helps ensure that figures meet PLOS requirements. To use PACE, you must first register as a user. Registration is free. Then, login and navigate to the UPLOAD tab, where you will find detailed instructions on how to use the tool. If you encounter any issues or have any questions when using PACE, please email PLOS at figures@plos.org. Please note that Supporting Information files do not need this step.

Attachment Submitted filename: NHANES .docx Click here for additional data file.

# Author comment: Author response to Decision Letter 0

**DOI:** 10.1371/journal.pone.0292810.r002

19 Sep 2023

Reviewer and Editor Comments

We thank the Reviewers and Editor for your comments. Please see our point by point responses below each of the Editor and Reviewer comments.

Editor Comments

1. Please ensure that your manuscript meets PLOS ONE's style requirements, including those for file naming. The PLOS ONE style templates can be found at

https://journals.plos.org/plosone/s/file?id=wjVg/PLOSOne_formatting_sample_main_body.pdf and

https://journals.plos.org/plosone/s/file?id=ba62/PLOSOne_formatting_sample_title_authors_affiliations.pdf

Response - We have revised the abstract and removed headings to meet the formatting requirements. Please let us know if we have missed something else.

2. Thank you for stating the following financial disclosure:

“The authors received no specific funding for this work. Institutional funding from York University was used to support this manuscript. York University did not have a role in the manuscript writing editing approval or decision to publish.”

At this time, please address the following queries:

a) Please clarify the sources of funding (financial or material support) for your study. List the grants or organizations that supported your study, including funding received from your institution.

b) State what role the funders took in the study. If the funders had no role in your study, please state: “The funders had no role in study design, data collection and analysis, decision to publish, or preparation of the manuscript.”

c) If any authors received a salary from any of your funders, please state which authors and which funders.

d) If you did not receive any funding for this study, please state: “The authors received no specific funding for this work.”

Please include your amended statements within your cover letter; we will change the online submission form on your behalf.

Response - We have revised the funding statement to: “The authors received no specific funding for this work.”

3. We noted in your submission details that a portion of your manuscript may have been presented or published elsewhere. [This manuscript or its essence has not been published as a peer-review but a prior version of this manuscript was published as part of Simone Daniels’ MSc thesis at York University. This manuscript includes additional data from the latest NHANES survey (2017-2018), and with differing analyses and results (https://yorkspace.library.yorku.ca/xmlui/bitstream/handle/10315/38812/Daniels_Simone_B_2021_Masters.pdf?sequence=2&isAllowed=y)] Please clarify whether this [conference proceeding or publication] was peer-reviewed and formally published. If this work was previously peer-reviewed and published, in the cover letter please provide the reason that this work does not constitute dual publication and should be included in the current manuscript.

Response - The thesis was not peer reviewed, and is published online by the university library. This is standard practice at most if not all Canadian Universities. Although the general topic is the same, the manuscript is substantially different from the thesis paper in its analyses and conclusions. Thus, we feel that this would not constitute dual publication as the data, results and figures differ.

4. We note that you have included the phrase “data not shown” in your manuscript. Unfortunately, this does not meet our data sharing requirements. PLOS does not permit references to inaccessible data. We require that authors provide all relevant data within the paper, Supporting Information files, or in an acceptable, public repository. Please add a citation to support this phrase or upload the data that corresponds with these findings to a stable repository (such as Figshare or Dryad) and provide and URLs, DOIs, or accession numbers that may be used to access these data. Or, if the data are not a core part of the research being presented in your study, we ask that you remove the phrase that refers to these data.

Response – We have removed that phrase in the manuscript and have added the results to the manuscript.

Review Comments to the Author

Reviewer #1: There are several language and grammar mistakes in the manuscript. A thorough editing is required. WL is the abbreviation mentioned for weight loss, though in some parts it is also used instead of WL exercises making the phrases confusing

Response – We apologize, but the abbreviation WL is only used in the abstract and tables/figures, and is meant to only mean weight loss. I have asked a colleague to edit, and have made several changes to hopefully improve the clarity of our writing. 

• In the abstract part: a sample size should be added.

Response – added.

• Various factors motivate individuals to get engaged in various WL plans, being obese alone is not necessarily the main factor. Body image also plays a critical role which has been neglected in this study. Moreover, underlying diseases are the motivation for such changes, these have also been ignored. In brief, the factors mentioned in this study are important but not sufficient alone. The authors should explain how they have managed to overcome these confounding factors, or else this study is biased.

Response – We complete agree with the reviewer that body image and weight loss intention likely play a large role in weight management behaviours. However, if you take the perspective of who should be prescribed weight loss, then guidelines normally suggest one of two approaches: 1) anyone over BMI of 30 kg/m2 or 2) BMI above 25 with comorbidities. Using approaches such as the Edmonton obesity staging system (EOSS), over 95% of individuals with a BMI greater than 30 kg/m2 have some sort of obesity related complication. Thus, our use of BMI alone would encompass the majority of individuals who would be prescribed weight loss in the clinical setting. We have added this omission to the discussion (page 15) and the limitations. 

Reviewer #2: 

As a descriptive article, it had acceptable analysis information, but it may be better to express the practical goals of this study more clearly.

Response – Thank you. We have revised the manuscript to more clearly express the study objectives at the end of the introduction. 

According to the fact that all the participants had informed consent for the use of their information from the beginning, it is reasonable to assume that there was no need for review by the ethics committee and the study is acceptable from the ethical point of view.

Response – Be assured that we followed the ethical guidelines in Canada. According to the Tri-Council Policy Statement: Ethical Conduct for Research Involving Humans (2022), Article 2.2:

“Research does not require REB review when it relies exclusively on information that is:

a. publicly available through a mechanism set out by legislation or regulation and that is protected by law; or

b. in the public domain and the individuals to whom the information refers have no reasonable expectation of privacy.“

Our research falls under point b: data in the public domain, and thus did not require REB review.

Attachment Submitted filename: Response to Reviewers.docx Click here for additional data file.

# Peer review: Decision Letter 1

**DOI:** 10.1371/journal.pone.0292810.r003

**Authors:** Patricia Khashayar

29 Sep 2023

Changes in the Prevalence of U.S. Adults using Diet, Exercise, Pharmaceuticals and Diet Products for Weight Loss Over Time: Analysis of NHANES 1999-2018.

PONE-D-23-14042R1

Dear Dr. Kuk,

We’re pleased to inform you that your manuscript has been judged scientifically suitable for publication and will be formally accepted for publication once it meets all outstanding technical requirements.

Within one week, you’ll receive an e-mail detailing the required amendments. When these have been addressed, you’ll receive a formal acceptance letter and your manuscript will be scheduled for publication.

An invoice for payment will follow shortly after the formal acceptance. To ensure an efficient process, please log into Editorial Manager at http://www.editorialmanager.com/pone/, click the 'Update My Information' link at the top of the page, and double check that your user information is up-to-date. If you have any billing related questions, please contact our Author Billing department directly at authorbilling@plos.org.

If your institution or institutions have a press office, please notify them about your upcoming paper to help maximize its impact. If they’ll be preparing press materials, please inform our press team as soon as possible -- no later than 48 hours after receiving the formal acceptance. Your manuscript will remain under strict press embargo until 2 pm Eastern Time on the date of publication. For more information, please contact onepress@plos.org.

Kind regards,

Patricia Khashayar

Academic Editor

PLOS ONE

Additional Editor Comments (optional):

Reviewers' comments:

Reviewer's Responses to Questions

**Comments to the Author**

1. If the authors have adequately addressed your comments raised in a previous round of review and you feel that this manuscript is now acceptable for publication, you may indicate that here to bypass the “Comments to the Author” section, enter your conflict of interest statement in the “Confidential to Editor” section, and submit your "Accept" recommendation.

Reviewer #1: All comments have been addressed

**********

2. Is the manuscript technically sound, and do the data support the conclusions?

The manuscript must describe a technically sound piece of scientific research with data that supports the conclusions. Experiments must have been conducted rigorously, with appropriate controls, replication, and sample sizes. The conclusions must be drawn appropriately based on the data presented. 

Reviewer #1: Yes

**********

3. Has the statistical analysis been performed appropriately and rigorously? 

Reviewer #1: Yes

**********

4. Have the authors made all data underlying the findings in their manuscript fully available?

The PLOS Data policy requires authors to make all data underlying the findings described in their manuscript fully available without restriction, with rare exception (please refer to the Data Availability Statement in the manuscript PDF file). The data should be provided as part of the manuscript or its supporting information, or deposited to a public repository. For example, in addition to summary statistics, the data points behind means, medians and variance measures should be available. If there are restrictions on publicly sharing data—e.g. participant privacy or use of data from a third party—those must be specified.

Reviewer #1: Yes

**********

5. Is the manuscript presented in an intelligible fashion and written in standard English?

PLOS ONE does not copyedit accepted manuscripts, so the language in submitted articles must be clear, correct, and unambiguous. Any typographical or grammatical errors should be corrected at revision, so please note any specific errors here.

Reviewer #1: Yes

**********

6. Review Comments to the Author

Please use the space provided to explain your answers to the questions above. You may also include additional comments for the author, including concerns about dual publication, research ethics, or publication ethics. (Please upload your review as an attachment if it exceeds 20,000 characters)

Reviewer #1: (No Response)

**********

7. PLOS authors have the option to publish the peer review history of their article (what does this mean?). If published, this will include your full peer review and any attached files.

If you choose “no”, your identity will remain anonymous but your review may still be made public.

**Do you want your identity to be public for this peer review?** For information about this choice, including consent withdrawal, please see our Privacy Policy.

Reviewer #1: **Yes: **Pouria Khashayar

**********

Attachment Submitted filename: Review final.docx Click here for additional data file.

# Editor report: Acceptance letter

**DOI:** 10.1371/journal.pone.0292810.r004

**Authors:** Patricia Khashayar

5 Oct 2023

PONE-D-23-14042R1 

Changes in the Prevalence of U.S. Adults using Diet, Exercise, Pharmaceuticals and Diet Products for Weight Loss Over Time: Analysis of NHANES 1999-2018. 

Dear Dr. Kuk:

I'm pleased to inform you that your manuscript has been deemed suitable for publication in PLOS ONE. Congratulations! Your manuscript is now with our production department. 

If your institution or institutions have a press office, please let them know about your upcoming paper now to help maximize its impact. If they'll be preparing press materials, please inform our press team within the next 48 hours. Your manuscript will remain under strict press embargo until 2 pm Eastern Time on the date of publication. For more information please contact onepress@plos.org.

If we can help with anything else, please email us at plosone@plos.org. 

Thank you for submitting your work to PLOS ONE and supporting open access. 

Kind regards, 

PLOS ONE Editorial Office Staff

on behalf of

Dr. Patricia Khashayar 

Academic Editor

PLOS ONE
